# Development of Delivery Systems with Prebiotic and Neuroprotective Potential of Industrial-Grade *Cannabis sativa* L.

**DOI:** 10.3390/molecules29153574

**Published:** 2024-07-29

**Authors:** Szymon Sip, Anna Stasiłowicz-Krzemień, Anna Sip, Piotr Szulc, Małgorzata Neumann, Aleksandra Kryszak, Judyta Cielecka-Piontek

**Affiliations:** 1 Department of Pharmacognosy and Biomaterials, Faculty of Pharmacy, Poznań University of Medical Sciences, Rokietnicka 3, 60-806 Poznań, Poland; szymonsip@ump.edu.pl (S.S.); astasilowicz@ump.edu.pl (A.S.-K.); 2 Department of Biotechnology and Food Microbiology, Poznań University of Life Sciences, Wojska Polskiego 48, 60-627 Poznań, Poland; anna.sip@up.poznan.pl; 3 Department of Agronomy, Poznań University of Life Sciences, Dojazd 11, 60-632 Poznań, Poland; piotr.szulc@up.poznan.pl (P.S.); malgorzata.neumann@up.poznan.pl (M.N.); 4 Department of Pharmacology and Phytochemistry, Institute of Natural Fibres and Medicinal Plants, Wojska Polskiego 71b, 60-630 Poznań, Poland; aleksandra.kryszak@iwnirz.pl

**Keywords:** *Cannabis Sativa*, SFE, antioxidant activity, neuroprotective activity, prebiotic, microbiome, industrial hemp

## Abstract

This study delves into the transformative effects of supercritical carbon dioxide (scCO_2_) cannabis extracts and prebiotic substances (dextran, inulin, trehalose) on gut bacteria, coupled with a focus on neuroprotection. Extracts derived from the Białobrzeska variety of Cannabis sativa, utilising supercritical fluid extraction (SFE), resulted in notable cannabinoid concentrations (cannabidiol (CBD): 6.675 ± 0.166; tetrahydrocannabinol (THC): 0.180 ± 0.006; cannabigerol (CBG): 0.434 ± 0.014; cannabichromene (CBC): 0.490 ± 0.017; cannabinol (CBN): 1.696 ± 0.047 mg/gD). The assessment encompassed antioxidant activity via four in vitro assays and neuroprotective effects against acetylcholinesterase (AChE) and butyrylcholinesterase (BChE). The extract boasting the highest cannabinoid content exhibited remarkable antioxidant potential and significant inhibitory activity against both enzymes. Further investigation into prebiotic deliveries revealed their proficiency in fostering the growth of beneficial gut bacteria while maintaining antioxidant and neuroprotective functionalities. This study sheds light on the active compounds present in the Białobrzeska variety, showcasing their therapeutic potential within prebiotic systems. Notably, the antioxidant, neuroprotective, and prebiotic properties observed underscore the promising therapeutic applications of these extracts. The results offer valuable insights for potential interventions in antioxidant, neuroprotective, and prebiotic domains. In addition, subsequent analyses of cannabinoid concentrations post-cultivation revealed nuanced changes, emphasising the need for further exploration into the dynamic interactions between cannabinoids and the gut microbiota.

## 1. Introduction

Alterations in gut microbiota are increasingly recognised as pivotal contributors to the pathophysiology of neurodegenerative diseases, specifically Alzheimer’s and Parkinson’s. In the context of Alzheimer’s disease, studies indicate a shift in gut microbiota composition, characterised by a decrease in beneficial bacterial species and an increase in pro-inflammatory bacteria [1,2,3]. This dysbiosis is associated with the accumulation of amyloid-beta plaques in the brain, a hallmark of Alzheimer’s pathology [4,5]. The gut–brain axis dysfunction in Alzheimer’s is implicated in neuroinflammatory responses and oxidative stress, further exacerbating cognitive decline [6,7]. In Parkinson’s disease, a distinct pattern of gut microbiota alterations emerges. The abundance of specific bacterial taxa, such as *Prevotellaceae* and *Lactobacillaceae*, appears to be reduced, while the presence of potentially pathogenic bacteria like *Enterobacteriaceae* increases. These microbial composition shifts correlate with alpha-synuclein aggregation, a key pathological feature of Parkinson’s [8,9]. The gut–brain axis disruption in Parkinson’s contributes to neuroinflammation, potentially facilitating the propagation of alpha-synuclein pathology from the gut to the brain [10]. The mechanistic links between gut microbiota changes and neurodegenerative diseases involve the production of neuroactive metabolites, modulation of the immune system, and the influence on the integrity of the gut epithelial barrier. Specific microbial metabolites, including short-chain fatty acids, neurotransmitters, and lipopolysaccharides, can enter systemic circulation and affect the central nervous system, contributing to neuroprotection and reduction in oxidative stress [11,12,13].

Understanding the dynamics of gut microbiota alterations in neurodegenerative diseases provides valuable insights into potential therapeutic targets. Strategies aimed at restoring a balanced and diverse gut microbiome, such as probiotics, prebiotics, and dietary interventions, hold promise for mitigating the progression of neurodegenerative disorders by modulating the gut–brain axis and addressing systemic inflammation [14,15].

*Cannabis sativa* boasts a rich history of medicinal use spanning centuries, harnessing a diverse array of bioactive compounds such as cannabinoids, terpenes, and flavonoids, each demonstrating notable health-promoting properties [16,17,18]. Cannabinoids, including the extensively studied non-psychoactive cannabidiol (CBD), interact with the human body’s endocannabinoid system (ECS). The ECS, a regulatory network, influences various physiological processes, prominently governing gut function. While the precise impact of cannabinoids on the gut microbiome is an ongoing area of investigation, the dynamic interplay between the gut microbiota and the ECS is increasingly recognised. This reciprocal relationship implies that alterations in the gut microbiome can influence ECS signalling and vice versa, thereby influencing gut health [19,20].

In the context of neurodegenerative diseases, the therapeutic potential of modulating the endocannabinoid system using cannabinoids has garnered significant attention. CBD, renowned for its neuroprotective effects, emerges as an up-and-coming candidate [21]. Its ability to safeguard the nervous system from damage or degeneration positions it as a potential therapeutic agent for conditions characterised by neurodegeneration. The well-known psychoactive cannabinoid tetrahydrocannabinol (THC) also interacts with the endocannabinoid system, impacting mood, perception, and cognitive functions [22]. While THC’s psychoactive nature is associated with the “high” from cannabis use, its potential therapeutic role in mitigating neurodegenerative processes warrants exploration [23]. Cannabinol (CBN), a by-product of THC degradation found in aged cannabis, presents intriguing possibilities for addressing sleep disorders, with some studies suggesting sedative effects [24,25]. Cannabichromene (CBC), a non-psychoactive cannabinoid, holds promise in anti-inflammatory, anti-depressant, and anti-cancer applications, expanding the scope of research in this area [26,27]. Cannabigerol (CBG), often called the “mother cannabinoid”, is gaining recognition for potential anti-inflammatory, antibacterial, and neuroprotective effects [28,29]. The modulation of the endocannabinoid system through cannabinoids offers a potential avenue for therapeutic interventions in neurodegenerative diseases. Utilising cannabinoids like CBD to influence the ECS may mitigate neuroinflammation, oxidative stress, and other pathological processes associated with conditions such as Alzheimer’s and Parkinson’s [30]. The intricate interplay between cannabinoids, the gut microbiota, and the endocannabinoid system thus represents a multifaceted area of research with implications for understanding and treating various neurodegenerative conditions.

The robust gut microbiome supports the immune system, enhancing its ability to fight pathogens and maintain health [31]. The prebiotic effect of *Cannabis sativa* can also contribute to a more substantial gut barrier, potentially reducing the risk of gastrointestinal issues. A well-functioning gut barrier helps prevent the leakage of harmful substances into the bloodstream and maintains a healthy gut lining [32,33]. This, in turn, supports the overall health of the digestive system. The diverse compounds in *Cannabis sativa* and prebiotic properties can foster beneficial gut bacteria growth [34]. A well-functioning gut barrier ensures that the integrity of the gut lining is maintained, reducing the risk of gastrointestinal issues. The combination of *Cannabis sativa* and prebiotics creates a symbiotic relationship that supports the flourishing of beneficial gut bacteria, fortifies the immune system, and reinforces the protective barrier of the gut [35,36]. Developing prebiotic systems represents a potent strategy for delivering plant-derived active compounds endowed with multifaceted effects. This approach assumes particular significance in neurodegenerative diseases, where the imperative for comprehensive therapeutic interventions is paramount.

Ongoing research explores using *Cannabis sativa* varieties characterised by low cannabinoid content, aiming to unlock their potential applications. Remarkably, extracts from the leaves of industrial varieties like Białobrzeska, Henola, and Tygra, traditionally cultivated for fibres and seeds, exhibited significant antioxidant potential. The study, employing ultrasound-assisted extraction, demonstrated the robust antioxidant capacity of these varieties, hinting at potential applications in medicine or targeted processing for active compound extraction [37]. Furthermore, investigations on the inflorescences of these *Cannabis sativa* varieties and extracts obtained using supercritical carbon dioxide (scCO_2_) extraction unveiled additional neuroprotective potential. The inhibition of acetylcholinesterase (AChE), butyrylcholinesterase (BChe), and tyrosinase indicated untapped avenues for obtaining active compounds. This sheds light on potential applications in various *Cannabis sativa* industry sectors and suggests novel development paths [38].

Prebiotics play a crucial role in modulating the gut microbiota, influencing various aspects of human health. Among the diverse compounds with potential prebiotic properties, dextran, inulin, and trehalose have garnered significant interest for their unique characteristics and potential health benefits.

A significant aspect of this study lies in its comprehensive approach, encompassing not only the analysis of cannabinoids derived from an industrially cultivated *Cannabis sativa* variety primarily intended for seed production but also the development of advanced delivery systems with prebiotic attributes. This innovative strategy aims to fully exploit the therapeutic potential of the *Cannabis sativa* variety by facilitating multifaceted actions. By integrating the investigation of cannabinoids from an industrially cultivated *Cannabis sativa* strain with the refinement of prebiotic delivery systems, this research introduces a holistic perspective to cannabis investigation. This approach transcends traditional boundaries, offering substantial potential in elucidating the reservoir of cannabinoids within *Cannabis sativa*, traditionally cultivated for seed production. Simultaneously, the study pioneers the development of sophisticated delivery systems with prebiotic potential, exemplifying a multifaceted approach to harnessing the complete therapeutic benefits of cannabinoids.

## 2. Results and Discussion

Our investigation commenced with the creation of a series of extracts derived from a specific raw material: the panicle of the *Cannabis sativa* variety *Białobrzeska*. This particular variety, classified as industrial *Cannabis sativa* and cultivated in Poland, is predominantly known for its fibre, which contributes to producing textiles, paper, and various industrial goods. Beyond its fibre content, the *Białobrzeska* variant yields seeds notably rich in protein and other essential nutrients, rendering them a sought-after ingredient in health foods and supplements. While recognised as an industrial *Cannabis sativa* variety, *Białobrzeska* typically maintains low levels of THC, the psychoactive cannabinoid associated with cannabis [39]. Nonetheless, it may contain other non-psychoactive cannabinoids, including CBD, CBG, and CBC. We meticulously delineated critical process conditions to procure the most potent extract, instrumental in the subsequent formulation of the prebiotic delivery system. These encompassed precise specifications for temperature, pressure, and the volume of CO_2_ employed in the extraction process, as outlined in Table 1. In-depth exploration of these critical parameters was integral to optimising the extraction process, ensuring the concentration of bioactive compounds conducive to our study’s objectives. As detailed in Table 1, the modifications applied aimed to enhance the extraction efficiency and enrich the resulting extract with targeted cannabinoids, laying the foundation for the subsequent phases of our investigation.

We used High-Performance Liquid Chromatography (HPLC) to carefully analyse the content of five necessary cannabinoids: cannabidiol (CBD), cannabigerol (CBG), cannabinol (CBN), tetrahydrocannabinol (THC), and cannabichromene (CBC) in all our extracted samples. This method allowed us to measure these cannabinoids accurately and their total amount, as shown in Table 2. HPLC is a precise tool that ensures we can detect even tiny amounts of these compounds. This thorough analysis is crucial for understanding the chemical makeup of the *Białobrzeska Cannabis sativa* variety extracts. By including the concentrations of each cannabinoid and their total sum, we obtain a detailed picture of what is in the extract. The results in Table 2 show that our extraction process worked well in concentrating these cannabinoids. This detailed profile gives us essential information about potentially using the *Białobrzeska Cannabis sativa* extracts, especially in developing prebiotic delivery systems.

Upon analysing the active compound content, extract 9 emerged as the most potent, displaying the highest concentrations, affirming the effectiveness of the defined critical parameters in the supercritical fluid extraction (SFE) process. Notably, the consistently low levels of THC across all extracts align with expectations for an industrial *Cannabis sativa* variety primarily cultivated for fibre production. Despite its industrial focus, the *Białobrzeska* variety showcases the presence of cannabinoids, indicating its potential not only for medicinal applications in varieties with high cannabinoid content but also for utilising by-products in food or textile production. This underscores industrial *Cannabis sativa*’s versatility, suggesting avenues for further processing to derive therapeutic compounds for medical applications. The detailed cannabinoid profiles presented in Table 2 provide a robust foundation for understanding the chemical composition of the extracts. The varying concentrations of CBD, CBG, CBN, THC, and CBC underscore the nuanced nature of *Białobrzeska* variety, presenting opportunities for tailored applications in different industries. These findings contribute to the broader discourse on harnessing the potential of industrial *Cannabis sativa* for diverse purposes, including medicine and sustainable material production.

**Figure 1:**
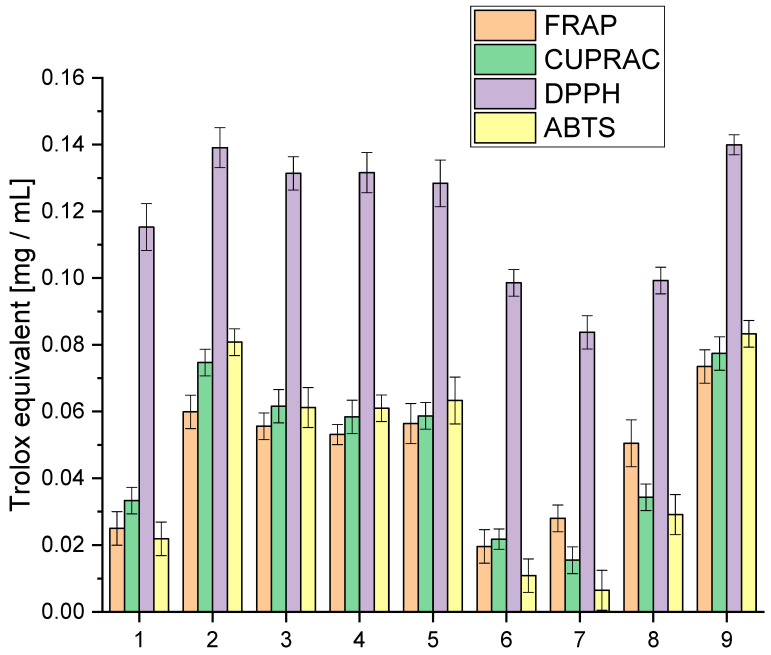
Analysis of the obtained extracts 1–9 antioxidant activity using the model FRAP, Cuprac, DPPH and ABTS expressed in Trolox equivalent.

We explored the entourage effect in plant raw materials, highlighting how different active compounds work together synergistically. This means that having a high concentration of one compound does not necessarily mean that it will have the most significant biological effect. Instead, it is the combination and balance of multiple compounds that matter. We conducted a detailed analysis of the extracts to understand their antioxidative activity using specific lab tests, as shown in Figure 1. This analysis aimed to uncover how the mixture of cannabinoids, terpenes, and other active compounds influences the overall antioxidative potential.

**Figure 2:**
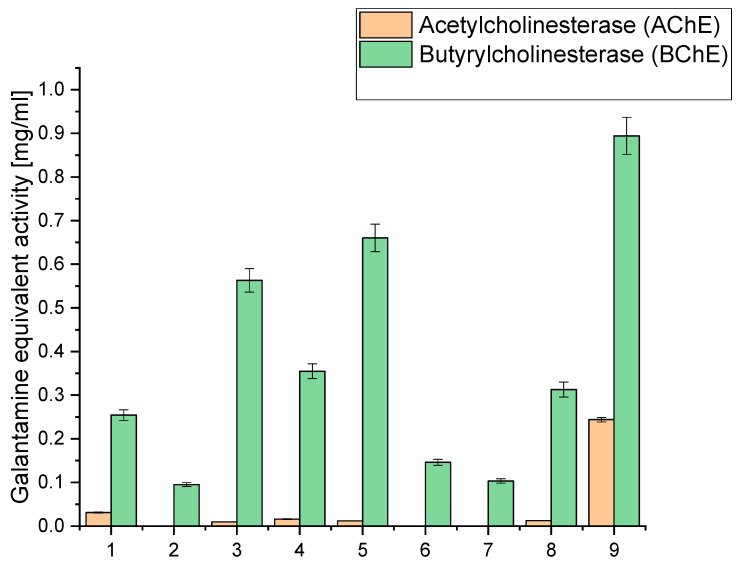
Analysis of acetyl- and butyrylcholinesterase inhibition of the obtained extracts 1–9 expressed as galanthamine equivalent.

Simultaneously, our study extended to evaluating acetyl- and butyrylcholinesterase inhibition (AChE and BChE), an essential facet in the quest for neuroprotective potential, as depicted in Figure 2. We sought to unravel the extracts’ potential in safeguarding neural functions by strategically directing our focus on these enzymatic activities. The numerical data of the obtained results of antioxidant and biological activity are included in the Supplementary Materials (Tables S2 and S3).

The analysis of antioxidant activity indicated that extract 9 was the most active in all the in vitro models used. All tested extracts were characterised by high activity in the DPPH model, giving activity at the Trolox equivalent level of 0.0837–0.13995 mg/mL. The highest activity variability was observed for the ABTS model, which occurred at the Trolox equivalent level of 0.0065–0.0833 mg/mL. In the case of the applied Cuprac and FRAP models, differences were observed at the Trolox equivalent level of 0.0155–0.0774 mg/mL and 0.0196–0.0735 mg/mL, respectively. The observed variability in activity underscores the intricate interplay of compounds within each extract, revealing a spectrum of antioxidant capacities. These findings not only position extract 9 as a standout performer but also emphasise the dynamic and versatile nature of the entire spectrum of extracts, fostering a nuanced comprehension of their antioxidant capabilities.

However, the cannabinoid content alone does not fully explain the antioxidant activity of the extracts. Other compounds, such as terpenes and flavonoids, also contribute significantly to the antioxidant properties. Terpenes, which include compounds like β-caryophyllene, myrcene, and limonene, can enhance the antioxidant capacity through synergistic effects, known as the entourage effect. This effect refers to the phenomenon where different compounds within the extract work together to produce a more robust antioxidant response than any single compound alone [40,41].

For extracts 6–8, the lower antioxidant activity might also be due to a suboptimal combination of cannabinoids and terpenes, which could result in weaker synergistic interactions. The lower amounts of CBG, known for its potent antioxidant properties, especially in extracts 6 and 7, could significantly impact their overall antioxidant capacity. Furthermore, the balance and interaction between various cannabinoids, such as the ratio of CBD to THC or the presence of CBN and CBC, play crucial roles in determining antioxidant efficacy.

The assessment of antioxidant activity is a complex issue and requires different models reflecting various mechanisms of action [42]. In our study, we observed significant differences in the activity of the extracts in four different assays: FRAP, Cuprac, DPPH, and ABTS. Such differences can be attributed to the diversity of active compounds in the extracts and the different mechanisms underlying the specific assay. It is well known that a range of active compounds in plant extracts contribute to their antioxidant properties. These include phenolic acids, flavonoids, and terpenoids, which exhibit varying antioxidant activity and can act through different mechanisms [43,44]. For example, phenolic acids such as caffeic acid and ferulic acid act as electron and hydrogen atom donors, while flavonoids such as quercetin and rutin scavenge free radicals through single electron transfer reactions [45]. Terpenoids, such as β-carotene and lycopene, act as singlet oxygen quenchers [46]. The distinctive activity profiles observed in our study find their roots in the diverse mechanisms underpinning the assays employed. Each assay, a unique window into the extract’s antioxidant potential, unveils specific facets of its functional dynamics. In the FRAP and Cuprac assays, the extracts’ prowess to reduce Fe(III) and Cu(II) ions takes centre stage, respectively. These assays delve into the extract’s capacity to donate electrons, offering insights into its electron transfer capabilities within distinct chemical contexts.

On the other hand, the DPPH and ABTS assays pivot on the extract’s ability to scavenge free radicals, a pivotal attribute in neutralising oxidative stress. Here, the focus shifts to the extract’s capacity to intercept and nullify these highly reactive species. The spectrum of active compounds within the extract exhibits varying degrees of efficacy, contingent upon the specific mechanism tested. The intricate interplay of cannabinoids, terpenes, and other bioactive compounds manifests diverse functional roles, shaping the extract’s overall antioxidant activity. Our study underscores the imperative of employing a battery of assays. This multifaceted approach not only elucidates the diverse antioxidant capacities of the extracts but also unravels the underlying mechanisms steering their activity. By embracing this comprehensive strategy, we navigate the complex landscape of antioxidant potential, laying the foundation for a more profound understanding of the extract” multifaceted contributions to oxidative stress mitigation.

Inhibition of acetylcholinesterase (AChE) and butyrylcholinesterase (BChE) stands as a pivotal strategy in neuroprotection, particularly concerning neurodegenerative diseases like Alzheimer’s disease (AD) [47]. Dysregulated cholinesterase activity, notably elevated AChE and BChE levels, is a hallmark of AD pathology, contributing to cognitive decline and memory impairment. The cholinergic hypothesis underscores the significance of cholinergic neurotransmission in AD pathogenesis, emphasising the role of AChE-mediated acetylcholine hydrolysis in cognitive deficits [48]. Therefore, inhibiting AChE activity represents a potential avenue for ameliorating symptoms and slowing disease progression. The neuroprotective effects of AChE and BChE inhibition stem from their ability to increase acetylcholine levels, thereby enhancing cholinergic neurotransmission and promoting neuronal survival. This mechanism is crucial for preserving cognitive function and attenuating neurodegeneration in AD. Clinical trials have demonstrated the efficacy of cholinesterase inhibitors such as donepezil, rivastigmine, and galantamine in improving cognitive function and delaying disease progression in AD patients. These drugs exert their effects by inhibiting AChE and BChE, leading to elevated acetylcholine levels in the brain [49,50,51].

Our study investigated the extract’s capability to inhibit AChE and BChE, critical enzymes involved in neurotransmission processes. AChE and BChE are integral to maintaining cognitive functions, as they regulate the levels of neurotransmitters in the brain. Inhibiting these enzymes is a sought-after strategy in neuroprotection, as it can modulate neurotransmitter levels and potentially mitigate cognitive decline. Our findings shed light on the neuroprotective potential of the extracts by demonstrating their ability to inhibit AChE and BChE activities. This comprehensive evaluation underscores the multifaceted contributions of the extracts to neuroprotection, offering promising avenues for further research in cognitive health and neurological well-being (Figure 2).

When examining the inhibition against AChE and BChE, we observed that extract 9 exhibited the highest AChE inhibition, with a value of 0.2438, equivalent to galantamine. This extract had a relatively high CBD content (6.675 mg/g) compared to the other extracts. Similarly, it displayed the highest BChE inhibition, with a value of 0.894, equivalent to galantamine. Notably, extract 9 had the highest sum of cannabinoids among all the extracts (9.475 mg/g), indicating a rich composition of active compounds. Extracts 3, 4, 5, and 8 also showed moderate AChE and BChE inhibition. These extracts also had notable CBD content, ranging from 5.065 mg/g to 6.071 mg/g. High CBD levels in these extracts suggest a potential correlation between CBD content and cholinesterase inhibitory activity. On the other hand, extracts 1, 2, 6, and 7 exhibited lower inhibitory activity against both AChE and BChE. These extracts had relatively lower cannabinoid content than the others, which might contribute to their reduced inhibitory effects.

The significantly higher cholinesterase inhibitory activities observed in extract 9 compared to extracts 6–8 can be linked to its higher total concentration of cannabinoids, as well as the presence and higher concentration of specific cannabinoids that may contribute more effectively to cholinesterase inhibition. Extract 9 contains 6.675 mg/g of CBD, 0.434 mg/g of CBG, 1.696 mg/g of CBN, 0.180 mg/g of THC, and 0.490 mg/g of CBC, summing up to a total of 9.475 mg/g of cannabinoids. The specific roles of these cannabinoids in cholinesterase inhibition are significant; for instance, CBN has been reported to have notable effects in such biochemical activities. The relatively lower inhibitory activities in extracts 6 and 7 can be attributed to their lower concentrations of cannabinoids and other active compounds. Extract 6 has a total of 3.484 mg/g of cannabinoids, while extract 7 has only 1.810 mg/g. The lower concentrations of these bioactive compounds directly correlate with their diminished ability to inhibit AChE and BChE.

Other bioactive compounds, such as terpenes and flavonoids, are crucial in cholinesterase inhibition. Terpenes, like α-pinene, limonene, and β-caryophyllene, may enhance the inhibitory effects through synergistic interactions with cannabinoids, contributing to the overall efficacy. The entourage effect, where different compounds in the extract work together to produce a more potent inhibitory response, is essential in understanding the varying degrees of activity among different extracts [41,52]. Extract 8, although having a higher concentration of cannabinoids (6.062 mg/g) than extracts 6 and 7, still demonstrates lower inhibitory activities than extract 9. This suggests that the total concentration and the specific composition and ratio of cannabinoids and other compounds influence the inhibitory efficacy. The balance between different cannabinoids and their interaction with terpenes might be less optimal in extract 8, resulting in reduced cholinesterase inhibition compared to extract 9.

While our initial findings are promising, they represent just the beginning of our exploration. We need more thorough and detailed studies to understand the specific relationships and mechanisms behind these inhibitory effects. It is essential to recognise that many factors, including other plant chemicals and how they interact, contribute to the overall activity of the extracts. As we continue to delve into this complex area of bioactivity, these early findings urge us to dig deeper. By unravelling the layers of complexity, we can better understand the precise mechanisms behind the extracts’ inhibitory abilities. This, in turn, will pave the way for more nuanced therapeutic applications and a deeper grasp of their potential for pharmacological use.

As observed in this study, the inhibitory activity of *Cannabis sativa* extracts against AChE and BChE aligns with previous research investigating the potential of *Cannabis sativa*-derived compounds in modulating these enzymes [53,54]. Various studies have explored the effects of *Cannabis sativa* extracts and individual cannabinoids on cholinesterase activity, providing valuable insights into their mechanisms of action.

Several cannabinoids found in *Cannabis sativa*, including delta-9-tetrahydrocannabinol (THC), cannabidiol (CBD), and cannabigerol (CBG), have been studied for their activities on AChE and BChE. THC has been shown to possess moderate inhibitory effects on these enzymes, suggesting its potential role in modulating cholinergic transmission [55]. However, THC is also known for its psychoactive properties, which may limit its widespread use for therapeutic purposes.

Conversely, CBD has been found to have minimal direct inhibitory effects on AChE and BChE [56]. Instead, CBD modulates these enzymes’ activity indirectly by influencing other molecular targets within the endocannabinoid system and other signalling pathways. CBD’s neuroprotective and anti-inflammatory properties have been proposed to contribute to its potential therapeutic effects on neurodegenerative diseases [57]. CBG, a minor cannabinoid found in *Cannabis sativa*, has shown promising inhibitory effects on AChE, suggesting its potential as a therapeutic agent for cholinergic dysfunction conditions. CBG has also demonstrated antioxidant and anti-inflammatory properties, highlighting its potential neuroprotective effects [58].

When these individual cannabinoids are combined in a supercritical *Cannabis sativa* extract, the entourage effect may come into play. The entourage effect suggests that the synergistic interactions between cannabinoids, terpenes, and other phytochemicals present in the extract can enhance their overall therapeutic potential [59,60]. While THC, CBD, and CBG may have their activities on AChE and BChE, their combined presence in a *Cannabis sativa* extract could lead to a more significant inhibitory effect on these enzymes than isolated cannabinoids alone. It is worth noting that the specific composition and ratios of cannabinoids and other bioactive compounds in a *Cannabis sativa* extract can vary depending on the extraction method and the strain of *Cannabis sativa* used [61,62]. Thus, the extent and nature of the entourage effect in inhibiting AChE and BChE may differ among different *Cannabis sativa* extracts.

However, it is essential to acknowledge the limitations of this study and the existing literature. The observed relationships between cannabinoid content and inhibitory activity are correlative and do not establish causation. Further mechanistic studies are required to elucidate the specific interactions between cannabinoids and cholinesterase enzymes. In conclusion, the findings from this study, combined with previous research, suggest that *Cannabis sativa* extracts and individual cannabinoids, particularly CBD, exhibit inhibitory activity against AChE and BChE. These results support the potential of *Cannabis sativa*-derived compounds as valuable candidates for developing cholinesterase inhibitors for various applications, including neurodegenerative diseases. Further research is warranted to understand these compounds’ underlying mechanisms and therapeutic potential fully.

Moving forward with our investigation, we delved into prebiotics, carefully designing systems infused with prebiotic potential. Through a meticulous lyophilisation process, we created three distinct systems tailored to leverage the established prebiotic properties of dextran, inulin, and trehalose. These well-known substances in prebiotic research were chosen for their unique characteristics. After thoroughly examining their prebiotic properties, we integrated them into our systems to explore their interaction with the complex gut microbiome.

Dextran, a polysaccharide produced by the fermentation of sucrose, has shown promising prebiotic effects. It acts as a substrate for beneficial gut bacteria, such as Bifidobacterium and *Lactobacillus*, stimulating their growth and activity. Dextran also exhibits potential immunomodulatory properties and contributes to maintaining intestinal barrier function [63]. Inulin, a fructan-type carbohydrate, has been widely recognised as a prebiotic compound. It undergoes fermentation in the colon, selectively promoting the growth of beneficial bacteria, particularly *Bifidobacteria*. Inulin has been associated with improved bowel regularity, increased calcium absorption, and potentially positive effects on lipid metabolism [64]. Trehalose, a disaccharide composed of two glucose molecules, has shown prebiotic potential by selectively promoting the growth of beneficial gut bacteria. It has been reported to enhance the growth of *Bifidobacterium* and *Lactobacillus* strains while inhibiting the proliferation of pathogenic bacteria. Trehalose also exhibits antioxidative properties and has been investigated for its potential neuroprotective effects [65].

The prepared systems (1-dextran, 2-inulin, 3-trehalose) were further evaluated to assess the content of active compounds (Table 3).

Additionally, the analysis of antioxidant activity and neuroprotective effects was repeated to estimate any potential changes resulting from the preparation process and the carrier substances’ inherent activity (Table 4 and Table 5).

Our comprehensive analysis yielded promising findings, demonstrating our tailored systems’ robustness in preserving active compounds’ content. This resilience extends to the meticulously introduced prebiotic matrices—dextran, inulin, and trehalose. Additionally, the levels of antioxidant activity, a critical metric of biological relevance, remained steadfast across all evaluated systems. These observations underscore the potential of our engineered constructs as carriers for active compounds while offering antioxidant benefits. These findings represent a significant advancement in our exploration, confirming the effectiveness of our tailored systems in retaining bioactive entities and sustaining beneficial biological activities.

In the subsequent phase of our investigation, we conducted a comprehensive analysis of acetylcholinesterase (AChE) and butyrylcholinesterase (BChE) activities within the meticulously engineered systems and their carriers—dextran, inulin, and trehalose. This examination aimed to elucidate the neuroprotective potential inherent in our tailored constructs. The detailed assessment of these enzymatic activities, as outlined in Table 5, provides a nuanced understanding of how our engineered matrices may impact neuroprotective pathways. The intricate interplay between the introduced prebiotic matrices and AChE’s and BChE’s enzymatic activities unveils potential neuroprotection implications (Table 5).

The data underscores that systems 1, 2, and 3 maintain the initial activity of the extract, affirming the preservation of neuroprotective potential within our engineered constructs. The not detected (Nd) status for prebiotic carriers indicates the absence of inhibitory effects, emphasising the selective neuroprotective influence of the formulated systems.

The prebiotic potential of the developed systems plays a crucial role in their application as functional ingredients. Evaluating the effectiveness of these systems in promoting the growth and activity of beneficial gut microbiota is essential for understanding their potential health benefits. This section presents the results of the prebiotic potential evaluation of the obtained systems (Table 6). These findings shed light on the ability of the developed systems to selectively support the growth of beneficial gut bacteria while inhibiting the proliferation of harmful species. Understanding the prebiotic potential of these systems is instrumental in harnessing their potential for improving gut health and overall well-being.

The results of this study shed light on the impact of different supercritical extract systems and excipients on the growth of specific bacterial strains over time. The population counts of *Faecalibacterium prausnitzii* DSM 107840 (P), *Bifidobacterium animalis* DSM 10140 (Ba), *Bifidobacterium longum subs. longum* DSM20219 (Bl), *Lactobacillus rhamnosus* GG ATCC 53103 (GG), *Lactobacillus helveticus* DSM 20075 (Lh), *Lactobacillus salivarius* LA 302 (Ls), and *Lactobacillus plantarum* 299v (9) were examined at four different time points (12 h, 24 h, 48 h, and 72 h).

Adding dextran as an excipient in the SFE+dextran system significantly affected the bacterial strains. It increased Ba and Bl population counts at all time points compared to the control. Specifically, at 12 h, the population counts of Ba and Bl were 2.27 × 10^7^ and 6.23 × 10^8^, respectively, in the SFE+dextran system, whereas in control, they were 7.24 × 10^7^ and 8.21 × 10^7^, respectively. This suggests that dextran positively influences the growth of these bacterial strains.

In contrast, the SFE+inulin system demonstrated variable effects on the bacterial strains. While it increased P and Bl population counts, it had a suppressive effect on the growth of Ba at 24 h. Specifically, at 24 h, the population counts of P and Bl in the SFE+inulin system were 4.94 × 10^8^ and 3.58 × 10^8^, respectively, while in control, they were 3.75 × 10^8^ and 1.64 × 10^8^, respectively. Interestingly, the SFE+inulin system exhibited a substantial increase in population counts of GG at 24 h and 48 h, with counts of 1.18 × 10^8^ and 1.44 × 10^9^, respectively, compared to the control counts of 4.92 × 10^7^ and 2.17 × 10^8^, indicating a potential prebiotic effect of inulin specifically on this strain.

The SFE+trehalose system also displayed diverse effects on the bacterial strains. It significantly enhanced the population counts of P at 12 h and 24 h and had a stimulatory effect on Bl at 12 h and 24 h. At 48 h and 72 h, the population counts of Ba and Bl in the SFE+trehalose system were considerably higher compared to the control. Specifically, at 48 h, the population counts of Ba and Bl in the SFE+trehalose system were 1.04 × 10^9^ and 1.17 × 10^9^, respectively, while in the control, they were 6.74 × 10^7^ and 1.36 × 10^8^, respectively. These results suggest that trehalose may act as a growth-promoting factor for these strains.

Comparing the effects of the excipients alone with their respective extract systems, dextran alone did not significantly impact bacterial growth. Inulin alone displayed a modest increase in the population counts of Bl at 24 h, with a count of 2.30 × 10^8^ compared to the control count of 3.75 × 10^8^. Trehalose alone exhibited slight growth-promoting effects on Ba and Bl at 72 h.

It is worth noting that the supercritical extracts from cannabis (SFE systems) have not been extensively studied regarding their influence on the gut microbiome [66]. The results of this study provide valuable insights into the potential effects of different supercritical extract systems and excipients on specific bacterial strains, highlighting their potential role in modulating the gut microbiome. Further optimisation of formulations, considering different combinations and concentrations, may enhance the prebiotic effects observed in this study. Analogous to the entourage effect observed in plant compounds, our findings suggest that the synergistic combination of *Cannabis sativa* extracts and prebiotic carriers contributes to enhanced prebiotic potential. The ability of formulated systems to modulate specific microbial strains suggests their potential to influence gut microbiota composition, with potential therapeutic implications. Future research could delve into understanding the molecular mechanisms underlying the observed prebiotic effects and exploring additional microbial strains for a more comprehensive assessment. Combining *Cannabis sativa* extracts and prebiotic carriers may offer synergistic benefits, acting as a dual-action system with prebiotic and bioactive properties. Consideration of interactions between *Cannabis sativa*-derived phytochemicals and microbial strains could provide insights into the multifaceted nature of prebiotic effects. This study establishes *Cannabis sativa* extracts as promising candidates for prebiotic applications, expanding the repertoire of prebiotic sources beyond traditional carbohydrates. Beyond gut health, the observed prebiotic potential may apply to functional foods, dietary supplements, and personalised nutrition. Using industrial *Cannabis sativa*, a sustainable crop, in prebiotic formulations aligns with the growing emphasis on environmentally friendly practices in the food and health industries. Acknowledging the specific limitations of this study, including the need for further clinical validation and exploration of diverse formulations, is essential for contextualising the findings. While some studies suggest cannabinoids can modulate the gut microbiome, the evidence is limited and often conflicting.

Additionally, most research has focused on specific cannabinoids like THC and CBD rather than supercritical extracts. Some evidence suggests that cannabinoids can exhibit antibacterial properties against specific pathogens, but their impact on beneficial probiotic bacteria is unclear [67]. Some studies have indicated that cannabinoids may affect the growth and viability of certain gut bacteria types, but more research is needed to understand the specific mechanisms and potential consequences. It is important to note that the effects of cannabis extracts on the gut microbiome can vary depending on factors such as dosage, frequency of use, and individual differences.

Table 7 presents the assessment of cannabinoid concentration reduction after 72 h of cultivation in various prebiotic systems. The percentage change in cannabinoid levels is documented for different time points and bacterial strains.

The results presented in Table 7 provide insights into the effect of different prebiotic systems on the reduction in cannabinoid concentrations after 72 h of cultivation. Each prebiotic system, represented by dextran, inulin, and trehalose, was assessed in conjunction with specific bacterial strains, including *Faecalibacterium prausnitzii*, *Bifidobacterium animalis*, *Bifidobacterium longum subs.longum*, *Lactobacillus helveticus*, *Lactobacillus rhamnosus GG*, *Lactobacillus salivarius*, and *Lactobacillus plantarum* 299v. The results demonstrate varying degrees of reduction in cannabinoid concentrations across different prebiotic systems and bacterial strains. For instance, in prebiotic system 1 (SFE extract + dextran), CBD concentrations were reduced by approximately 3.23%, 2.56%, and 4.89% in the presence of *Faecalibacterium prausnitzii*, *Bifidobacterium animalis*, and *Bifidobacterium longum subs.longum*, respectively. Similar trends were observed for other cannabinoids, such as CBG, CBN, THC, and CBC. Interestingly, prebiotic system 2 (SFE extract + inulin) exhibited different patterns of reduction in cannabinoid concentrations compared to prebiotic system 1. For instance, CBD concentrations in the presence of *Faecalibacterium prausnitzii*, *Bifidobacterium animalis*, and *Bifidobacterium longum subs.longum* were reduced by approximately 2.56%, 4.45%, and 2.01%, respectively.

Overall, these results suggest that the choice of prebiotic system and bacterial strain can influence the reduction in cannabinoid concentrations. Further investigation is warranted to elucidate these observations’ underlying mechanisms and determine the potential therapeutic application implications. Additionally, exploring a broader range of prebiotic systems and bacterial strains may provide further insights into optimising cannabinoid delivery and enhancing their therapeutic efficacy.

These findings underscore the dynamic process of cannabinoid degradation within prebiotic systems and suggest potential implications for the bioavailability of cannabinoids in the gastrointestinal tract. Further investigations are warranted to elucidate the specific mechanisms behind these observed reductions and to assess their impact on the overall therapeutic potential of cannabinoid-rich *Cannabis sativa* extracts. While the reductions in cannabinoid concentrations are statistically significant, they are relatively small. However, these reductions’ consistent and observable nature suggests a probable partial metabolism by the bacterial strains present in the prebiotic systems.

These preliminary results prompt the need for more extensive research to delve into the nuanced interactions between cannabinoids and gut microbiota. The subtle yet consistent changes observed suggest a complex interplay between cannabinoids and the microbial community, potentially resulting in metabolites with altered bioactivity. Therefore, future investigations should aim to unravel the specific pathways involved in the microbial metabolism of cannabinoids, providing valuable insights into their fate and potential transformation within the gut ecosystem.

## 3. Materials and Methods

### 3.1. Plant Material

The plant material employed in this investigation was sourced from the Białobrzeskie variety, generously provided by the Experimental Station for Cultivar Testing in Chrząstowo, affiliated with the Research Centre for Cultivar Testing in Słupia Wielka (Poland). The rotational cropping method was used in cultivation. In the previous year, sugar beet was grown as the predominant crop for *Cannabis sativa* in 2022. Following the recommended agricultural procedures, a series of tillage operations were conducted, including winter ploughing on 29 October 2021, harrowing with a spear on 17 March 2022, cultivation on 6 May 2022, and sowing on 9 May 2022. After hemp sowing, on 10 May 2022, the Boxer 800 EC herbicide was applied at 2.6 L per hectare.

Mineral fertilisation involved the application of Lubofos 12 (200 kg/ha), potassium salt (183 kg/ha), enriched superphosphate (115 kg/ha), urea (159 kg/ha), and salmag (119 kg/ha). The soil on the experimental field was classified as IIIa, complex 2, with the top horizons characterised as loamy sands, comprising 4% clay, 14% silt, and 83% sand fractions. The eluvial level exhibited slightly lower clay and silt fractions, while the enrichment (B) and bedrock levels were more compact. The pH value in the aqueous extract was 6.80*;* in KCl, it was approximately 0.5 units lower, falling within the upper range of slightly acidic conditions. The organic carbon content was around 1%, corresponding to a humus content of 1.7%. The total nitrogen content was 0.086%, with a C:N ratio of approximately 12:1.

Throughout the growing season, the thermal and moisture conditions proved favourable for the optimal growth and development of cannabis.

### 3.2. Reagents and Materials

Standard compounds (CBD, CBG, THC, CBC, and CBN) used in the HPLC analysis were supplied by Sigma-Aldrich (St. Louis, MO, USA). Trifluoric acid and acetonitrile (HPLC grade) were provided by Merck (Darmstadt, DE). High-quality pure water was prepared using a Direct-Q 3 UV purification system (Millipore, Molsheim, FR; model Exil SA 67120).

The prebiotic carriers (dextran (MW. 40,000), inulin, and trehalose) were obtained from Sigma Aldrich Chemie (Berlin, Germany).

Reagents used in the analysis of antioxidant and biological activity are as follows: 2,2-Diphenyl-1-picrylhydrazyl; iron (III) chloride hexahydrate; 2,2′-azino-bis(3-ethylbenzothiazoline-6-sulfonic acid); neocuproine, 2,4,6-Tri(2-pyridyl)-s-triazine; Trolox, supplied by Sigma-Aldrich (Schnelldorf, Germany). Sodium chloride and sodium hydrogen phosphate were purchased from Avantor Performance Materials (Gliwice, Poland). Ammonium acetate (NH_4_Ac) and methanol were supplied by Chempur (Piekary Śląskie, Poland). Cupric chloride dihydrate, ethanol (96%), isopropanol (99%), acetic acid (99.5%), sodium acetate trihydrate, and were obtained from POCH (Gliwice, PL).

For the microbiological studies, the following strains were assessed:

1. P—Faecalibacterium prausnitzii DSM 107840;
2. Ba—Bifidobacterium animalis DSM 10140;
3. Bl—Bifidobacterium longum subs.longum DSM20219;
4. Lh—Lactobacillus helveticus DSM 20075;
5. GG—Lactobacillus rhamnosus GG ATCC 53103;
6. Ls—Lactobacillus salivarius LA 302;
7. 9—Lactobacillus plantarum 299v.

All strains were stored in a Cryobank (Bacteria storage system, MAST Diagnosti-ca) at −20 °C. Before the studies, the strains were defrosted and passaged twice for re-generation in MRS broth (OXOID), the standard medium for Lactobacillus bacteria. Cultures were incubated/carried out at 37 °C for 24 h under anaerobic conditions.

The organic solvent evaporation process was carried out using incubator MaxQ 4450 (Thermo Scientific, Waltham, MA, USA). Qualitative and quantitative research used the high-performance liquid chromatograph Prominance-I LC-2030C and a UV detector. A plate reader (Multiskan GO (Thermo Scientific), and a laboratory incubator (MaxQ 4450, Thermo Scientific). A Radwag AS 220.X2 (Radom, Poland) analytical balance was used throughout the study to measure weight.

### 3.3. Preparation of scCO_2_ Cannabis Extracts

The extraction process was carried out analogously to the previous process with minor changes [68]. The extraction process employed the SFT-120XW apparatus with a 5 mL extraction vessel, utilising class 2 CO_2_ heated to 30 °C. The extraction occurred at vessel temperatures of 30–50 °C and a pressure of 3000–5000 psi in dynamic mode with a continuous flow of scCO_2_. After pumping 100–300 mL of the extractant, the process concluded with a heated collection line at 75 °C to prevent CO_2_ freezing. An amount of 5.0 g of pre-milled *Cannabis sativa* raw material was loaded into the extraction vessel compacted to reduce dead volumes. The vessel was heated without CO_2_, and then CO_2_ was introduced, gradually reaching the desired pressure. After stabilisation, the pre-heated collection line was opened, and the extract was collected in a 50 mL Falcon tube. This precise extraction aimed to optimise bioactive compound yield from the *Cannabis sativa* material.

### 3.4. Extract Purification

The extract obtained from scCO_2_ was subjected to winterization to remove the insoluble fractions, which could disturb the activity tests. For this purpose, the extract was dissolved in 50 mL of 96% ethanol and thoroughly mixed using a vortex to bring the active compounds into the solution. Then, the sample was placed in a freezer at −25 °C for 72 h. After this time, the sample was filtered under reduced pressure to obtain a clear solution containing a wax-free extract. The solution prepared this way was further used in the content analysis and activity studies. The obtained solutions were stored in tightly closed Falcon tubes at −25 °C during the research.

### 3.5. HPLC Method

For the quantitative analysis of cannabinoids, an isocratic HPLC method was meticulously developed using a Nexera apparatus (Shimadzu) equipped with a DAD detector. The method employed a CORTECS Shield RP18 2.7 μm, 4.6 mm × 150 mm (Waters) column. The mobile phase consisted of 0.1% trifluoroacetic acid (A) and acetonitrile (B), with a carefully optimised A:B = 41:59 ratio and a consistent flow rate of 2 mL/min. Throughout the analysis, the column was precisely thermostated at 35 °C. Injections of 10 µL were made, and detection was carried out at 228 nm. The determination of the amount of cannabinoids was made based on standard curves obtained from previously prepared solutions of pure cannabinoids; the presence was confirmed based on the retention time and the spectrum of the active compound in order to confirm their presence in the tested extracts. The analytical procedure spanned 50 min to ensure comprehensive cannabinoid profiling. For a more comprehensive understanding of the HPLC analysis, validation parameters, detailed calibration curves for each standard, and sample chromatographs have been thoughtfully compiled and provided in the Supplementary Materials (Table S1 and Figures S1 and S2).

HPLC analysis for samples after culture was performed by primary centrifugation of the biomass; then, the sample was filtered through a 0.22 μm nylon syringe filter.

### 3.6. Antioxidant Assays

Ferric Reducing Antioxidant Power (FRAP), Cupric Reducing Antioxidant Capacity (CUPRAC), 2,2-difenyl-1-pikrylohydrazyl (DPPH), and 2,2′-azino-bis(3-ethylbenzothiazoline-6-sulfonic acid) (ABTS) assays were determined according to the previously reported methods and presented as the Trolox equivalent calculated from the standard curve [38,69]:

#### 3.6.1. ABTS Assay

The ABTS cation radical was generated by potassium persulfate-induced electron loss from ABTS nitrogen atoms. Subsequently, a pre-formed radical cation was mixed with antioxidants, reducing ABTS radicals to their colourless, neutral form. HES concentrations in DMSO were prepared for the assay. In a 96-well plate, 50.0 µL of HES solutions and 200.0 µL of ABTS•+ solution per well were incubated with shaking for 10 min at room temperature. Absorbance was measured at λ = 734 nm post-incubation. ABTS scavenging activity was calculated using the formula:

ABTS scavenging activity (%) = (A0 − A1)/A0 × 100%.(1)

where:

A1—the absorbance of the test sample;
A0—the absorbance of control.

Trolox was served as the standard (Figure S3).

#### 3.6.2. CUPRAC Assay

In the CUPRAC method, antioxidants’ phenolic groups undergo oxidation to quinones, which in turn reduce the neocuproine–copper(II) complex to neocuproine–copper(I), resulting in a colour change from bluish to yellow [40]. Specifically, 50.0 µL of the extract or Trolox solution was combined with 150.0 µL of the CUPRAC reagent in a plate, followed by a 30 min incubation at room temperature in darkness [39]. Absorbance readings were then taken at 450 nm using a plate reader (Multiskan GO, Thermo Fisher Scientific, Waltham, MA, USA). The absorbance of the control and the extracts’ own absorbance was also compared, and the results were expressed as Trolox equivalents (Figure S4).

#### 3.6.3. DPPH Assay

The DPPH assay was conducted in a 96-well plate using spectrophotometry. The primary reagent used was a 0.2 mM methanol solution of DPPH. An amount of 25.0 µL of the extracts or Trolox solution was added to 175.0 µL of the DPPH solution to initiate the reaction. The plate was then incubated in darkness at room temperature for 30 min with shaking. Absorbance readings were taken at 517 nm using a plate reader (Multiskan GO, Thermo Fisher Scientific, Waltham, MA, USA). The absorbance of the blank (a mixture of DPPH solution and solvent) was also measured at 517 nm. Each sample’s absorbance at 517 nm was recorded. The percentage inhibition of DPPH radicals by the extracts or Trolox was calculated using the provided formula:

DPPH scavenging activity (%) = (A0 − A1)/A0 × 100%(2)

where:

A1—the absorbance of the test sample;
A0—the absorbance of control.

The results were expressed as Trolox equivalents (Figure S5).

#### 3.6.4. FRAP Assay

The FRAP assay involved mixing tested extracts with FRAP reagent and incubating the mixture at 37 °C. Absorbance was read at 593 nm, and the results were expressed as Trolox equivalents (Figure S6).

### 3.7. Neuroprotective Assays

The previously reported method determined AChE and BChE inhibition [70]. The activity was then converted to galanthamine equivalent using the standard curve for AChE and BChE (Figures S7 and S8). This method involves the use of artificial substrates, known as thiocholine esters. Thiocholine is released during enzymatic reactions with 5,5′-dithio-bis-(2-nitrobenzoic) acid (DTNB), resulting in the formation of the 3-carboxy-4-nitrothiolate anion (TNB anion). The degree of inhibition of AChE and BChE was determined by measuring the increase in thiocholine colour in a 96-well plate. The percentage of inhibition for AChE and BChE by the samples was calculated using the formula:

AChE/BChE inhibition (%) = 1 − (A1 − A1b)(A0 − A0b) × 100%(3)

where:

A1—the absorbance of the test sample;
A1b—the absorbance of the blank of the test sample;
A0—the absorbance of control;
A0b—the absorbance of the blank of control.

### 3.8. Delivery System Preparation

Preparation of the system involved the supercritical fluid extraction of *Cannabis sativa* following winterisation. The extract was then subjected to solvent evaporation using a vacuum evaporator to remove organic solvents. Subsequently, an ultrasonic bath prepared dextran, inulin, and trehalose solutions in flat-bottomed conical flasks at a concentration of 10% in an amount of 100 mL. After dissolving in water and obtaining a clear solution, each solution was added to an equal part of the evaporated *Cannabis sativa* extract in a 1:1 ratio based on the amount of raw material used for extraction. The mixture was then placed in a rotary incubator at a speed of 200 RPM for 1 h to ensure complete suspension of the extract on the prebiotic substances. The suspensions were poured onto trays and placed in a freezer at −30 °C overnight to freeze the mixtures fully. The prepared trays were placed in a Lyoquest −85 freeze dryer (Telstar, Eden Prairie, MN, USA). The lyophilisation process was carried out for 5 days at a temperature of −85 °C and constant pressure of 0.2 mPa to ensure complete drying of the systems. After lyophilisation, the systems were removed from the shelves and homogenised using an agate mortar to obtain a uniform mass. During the research, the systems were stored in tightly sealed Falcon tubes at a temperature of 8 °C.

### 3.9. Prebiotic Potencial

Regenerated probiotic strain cultures were centrifuged at 4500× *g* for 10 min, and the cells were washed with sterile physiological saline. The cell suspensions were then adjusted to 0.5 on the McFarland scale (equivalent to 1.5 × 10^8^ CFU/mL) using a McFarland Densitometer (Biosan). These suspensions were used to inoculate 2 mL of standard MRS broth medium (control) and MRS medium containing additional components: dextran, inulin, or trehalose as single-component and prebiotic systems 1–3. As additional excipients, these components were added to the MRS medium at a 1% mass ratio. The initial bacterial concentration in all media was adjusted to 1.5 × 10^5^ CFU/mL. Incubation was carried out without pH regulation at 37 °C for 24 h. After incubation, viable cell counts were determined using Koch’s plate method. Samples were decimally diluted in sterile physiological saline, plated on Petri dishes containing MRS agar (OXOID), and then incubated at 37 °C for 48–72 h. An automatic colony counter (Easy Count 2) was used to enumerate colonies and calculate viable cell numbers expressed in CFU/mL. The strain showing the best response to the tested systems was cultured on a 200.0 mL scale for 96 h under the same conditions. During incubation, samples were taken at 12, 24, 48, and 72 h to evaluate viable cell counts, reported as colony-forming units (CFU/mL). Each test was performed in triplicate.

### 3.10. Statistical Analysis

Statistica 13.3 software (StatSoft Poland, Krakow, Poland) was utilised for the statistical analysis. The experimental data underwent skewness and kurtosis tests to assess the normality of distribution, while Levene’s test was employed to evaluate the equality of variances. Statistical significance was determined using one-way analysis of variance (ANOVA), followed by the Bonferroni post hoc test for comparisons between experimental results in antioxidant and anticholinesterase assays. For prebiotic potential studies, statistical analysis was conducted separately for each time point and each strain, comparing the results of each system or probiotic to the control (0). Differences were considered significant at *p* < 0.05.

## 4. Conclusions

This study introduces an innovative approach to the development of prebiotic delivery systems for Cannabis sativa extracts, with a focus on the industrially relevant Białobrzeska variety. Our investigation explores the therapeutic potential of industrial hemp varieties conventionally utilised for fibre production.

Quantification of cannabinoid concentrations in the extracts unveiled significant levels of cannabidiol (CBD: 6.675 mg/g), tetrahydrocannabinol (THC: 0.180 mg/g), cannabigerol (CBG: 0.434 mg/g), cannabichromene (CBC: 0.490 mg/g), and cannabinol (CBN: 1.696 mg/g). These concentrations underscore the richness of bioactive compounds inherent in the Białobrzeska variety. Furthermore, assessment of antioxidant activity via four in vitro assays revealed the extracts’ noteworthy antioxidant potential. Additionally, we evaluated their neuroprotective effects against AChE and BChE, with the extract exhibiting the highest cannabinoid content demonstrating significant inhibitory activity against both enzymes, indicating its potential neuroprotective efficacy.

Moreover, we investigated the prebiotic properties of dextran, inulin, and trehalose carriers in fostering the growth of beneficial gut bacteria. Interestingly, these carriers sustained antioxidant and neuroprotective functionalities while promoting the growth of beneficial gut bacteria. Our observations revealed statistically significant reductions in cannabinoid concentrations after 72 h of cultivation within the prebiotic systems. While these reductions were modest, they underscore the intricate interplay between Cannabis sativa extracts and the gut microbiota, suggesting a potential partial metabolism of cannabinoids by microbial strains. These findings prompt inquiries into cannabinoid bioavailability and transformation in the gastrointestinal tract, warranting further investigations into the underlying mechanisms and broader therapeutic implications. Our comprehensive analysis highlights the promise held by by-products from industrial production as a source of active compounds.

## Figures and Tables

**Table 1:** Process conditions used in the SFE used for obtaining 9 extracts.

Extract	Temperature [°C]	Pressure [psi/bar]	CO_2_ Volume [mL]
1	50	5000/344.738	100
2	70	4000/275.790	100
3	50	3000/206.843	300
4	30	5000/344.738	200
5	30	4000/275.790	300
6	70	3000/206.843	200
7	30	3000/206.843	100
8	50	4000/275.790	200
9	70	5000/344.738	300

**Table 2:** The results of determining the content of active compounds using the HPLC method of obtained extracts 1–9.

	CBD	CBG	CBN	THC	CBC	Sum of Cannabinoids
Extract	[mg/gD *]	[mg/gD *]	[mg/gD *]	[mg/gD *]	[mg/gD *]	[mg/gD *]
1	3.078 ± 0.077	0.092 ± 0.003	0.500 ± 0.014	0.057 ± 0.002	0.145 ± 0.005	3.872 ± 0.107
2	1.157 ± 0.029	0.049 ± 0.002	0.275 ± 0.008	0.026 ± 0.001	0.082 ± 0.003	1.589 ± 0.044
3	5.097 ± 0.127	0.173 ± 0.005	0.863 ± 0.024	0.11 ± 0.004	0.323 ± 0.011	6.566 ± 0.181
4	5.300 ± 0.132	0.161 ± 0.005	0.529 ± 0.015	0.071 ± 0.002	0.342 ± 0.012	6.404 ± 0.176
5	6.071 ± 0.151	0.245 ± 0.008	1.034 ± 0.028	0.131 ± 0.005	0.350 ± 0.012	7.831 ± 0.216
6	2.574 ± 0.064	0.127 ± 0.004	0.556 ± 0.015	0.057 ± 0.002	0.169 ± 0.006	3.484 ± 0.096
7	1.335 ± 0.033	0.066 ± 0.002	0.282 ± 0.008	0.034 ± 0.001	0.093 ± 0.003	1.810 ± 0.050
8	5.065 ±0.126	0.145 ± 0.005	0.460 ± 0.013	0.056 ± 0.002	0.336 ± 0.011	6.062 ± 0.167
9	6.675 ±0.166	0.434 ± 0.014	1.696 ± 0.047	0.180 ± 0.006	0.490 ± 0.017	9.475 ± 0.261

gD *—dry mass of plant material.

**Table 3:** The results of determining the content of active compounds using the HPLC method in obtained systems 1–3.

	CBD	CBG	CBN	THC	CBC	Sum of Cannabinoids
System	[mg/gDs *]	[mg/gDs*]	[mg/gDs *]	[mg/gDs *]	[mg/gDs *]	[mg/gDs *]
1	6.665 ± 0.106	0.414 ± 0.011	1.691 ± 0.027	0.180 ± 0.004	0.478 ± 0.014	9.428 ± 0.231
2	6.666 ± 0.161	0.431 ± 0.010	1.690 ± 0.045	0.178 ± 0.003	0.488 ± 0.015	9.453 ± 0.222
3	6.668 ± 0.145	0.427 ± 0.017	1.688 ± 0.044	0.175 ± 0.005	0.477 ± 0.017	9.435 ± 0.258

gD *—a dry mass of the system.

**Table 4:** Numerical data for the antioxidant activity of the obtained systems 1–3 and prebiotic carriers. Means with the same superscript letters (a) within the same column do not differ significantly (*p* < 0.05).

System	FRAP	Std	CUPRAC	Std	DPPH	Std	ABTS	Std
	Trolox equivalent [mg/mL]							
1	0.0745 ^a^	0.009	0.0770 ^a^	0.006	0.1389 ^a^	0.002	0.0827 ^a^	0.002
2	0.0738 ^a^	0.008	0.0757 ^a^	0.005	0.1394 ^a^	0.005	0.0824 ^a^	0.003
3	0.0740 ^a^	0.008	0.0761 ^a^	0.007	0.1379 ^a^	0.004	0.0819 ^a^	0.008
Dextran	Nd *	-	Nd *	-	Nd *	-	Nd *	-
Inulin	Nd *	-	Nd *	-	Nd *	-	Nd *	-
Trehalose	Nd *	-	Nd *	-	Nd *	-	Nd *	-

* Nd—not detected.

**Table 5:** Numerical data for the neuroprotective activity of the obtained systems 1–3 and prebiotic carriers. Means with the same superscript letters (a) within the same column do not differ significantly (*p* < 0.05).

System	AchE	Std	BChE	Std
	Galantamine equivalent [mg/mL]			
1	0.2427 ^a^	0.00510	0.8925 ^a^	0.0404
2	0.2433 ^a^	0.00544	0.8968 ^a^	0.0415
3	0.2431 ^a^	0.00491	0.8935 ^a^	0.0431
Dextran	Nd *	-	Nd *	-
Inulin	Nd *	-	Nd *	-
Trehalose	Nd *	-	Nd *	-

* Nd—not detected.

**Table 6:** Results of prebiotic potential evaluation of the obtained systems 1–3 and prebiotic carriers in 72 h culture with 4 sampling points. Means with (*) within the same column within the same time point differ significantly from control (*p* < 0.05).

	P *	Ba *	Bl *	GG *	Lh *	Ls *	9 *
12 h/0 **	2.27 × 10^7^ ± 4.49 × 10^5^	6.68 × 10^8^ ± 5.03 × 10^6^	6.23 × 10^8^ ± 4.47 × 10^6^	7.15 × 10^7^ ± 3.17 × 10^6^	7.24 × 10^7^ ± 3.77 × 10^6^	8.21 × 10^7^ ± 3.03 × 10^6^	6.62 × 10^8^ ± 3.12 × 10^7^
24 h/0	5.08 × 10^7^ ± 2.54 × 10^5^	4.94 × 10^8^ ± 4.94 × 10^6^	3.58 × 10^8^ ± 3.49 × 10^6^	2.17 × 10^8^ ± 2.12 × 10^6^	3.75 × 10^8^ ± 3.68 × 10^6^	1.64 × 10^8^ ±1.61 × 10^6^	9.08 × 10^8^ ± 9.08 × 10^6^
48 h/0	2.60 × 10^7^ ± 1.30 × 10^5^	3.41 × 10^8^ ± 1.70 × 10^6^	2.38 × 10^8^ ± 1.19 × 10^6^	1.68 × 10^8^ ± 8.40 × 10^5^	1.30 × 10^8^ ± 6.50 × 10^5^	2.25 × 10^7^ ± 1.13 × 10^5^	8.08 × 10^8^ ± 8.08 × 10^6^
72 h/0	1.63 × 10^6^ ± 5.35 × 10^4^	3.08 × 10^8^ ± 1.01 × 10^6^	1.05 × 10^8^ ± 3.44 × 10^5^	8.21 × 10^7^ ± 2.68 × 10^5^	2.19 × 10^7^ ± 7.14 × 10^4^	1.11 × 10^7^ ± 3.57 × 10^4^	7.59 × 10^8^ ± 7.59 × 10^6^
12 h/1 **	4.07 × 10^7^ * ± 8.15 × 10^4^	2.56 × 10^8^ * ± 5.12 × 10^5^	1.44 × 10^8^ * ± 2.88 × 10^5^	1.75 × 10^8^ * ± 3.51 × 10^5^	3.39 × 10^7^ ± 6.79 × 10^4^	2.07 × 10^8^ * ± 4.14 × 10^5^	5.04 × 10^8^ * ± 1.01 × 10^6^
24 h/1	5.82 × 10^7^ ± 2.54 × 10^5^	6.56 × 10^8^ * ± 2.87 × 10^6^	1.44 × 10^9^ * ± 6.30 × 10^6^	1.18 × 10^8^ * ± 5.13 × 10^5^	4.92 × 10^7^ * ± 2.15 × 10^6^	2.17 × 10^8^ * ±9.43 × 10^5^	8.01 × 10^8^ * ± 4.01 × 10^6^
48 h/1	6.74 × 10^7^ * ± 3.37 × 10^5^	1.04 × 10^9^ * ± 5.20 × 10^6^	1.17 × 10^9^ * ± 5.85 × 10^6^	1.37 × 10^8^ * ± 6.85 × 10^5^	5.53 × 10^7^ * ± 2.77 × 10^6^	1.36 × 10^8^ * ± 6.81 × 10^5^	3.93 × 10^8^ * ± 3.94 × 10^6^
72 h/1	2.32 × 10^7^ * ± 1.16 × 10^5^	3.17 × 10^8^ ± 1.58 × 10^6^	2.40 × 10^8^ * ± 1.20 × 10^6^	5.12 × 10^7^ * ± 2.56 × 10^5^	5.92 × 10^7^ * ± 2.96 × 10^5^	1.19 × 10^8^ * ± 5.99 × 10^5^	2.40 × 10^8^ * ± 1.20 × 10^6^
12 h/2 **	4.25 × 10^6^ * ± 5.25 × 10^4^	1.06 × 10^9^ * ± 1.31 × 10^6^	7.90 × 10^8^ * ± 9.75 × 10^5^	1.51 × 10^8^ * ± 1.85 × 10^5^	5.16 × 10^8^ * ± 6.30 × 10^5^	4.96 × 10^8^ * ± 6.05 × 10^5^	2.11 × 10^8^ * ± 2.55 × 10^5^
24 h/2	6.31 × 10^6^ * ± 7.85 × 10^4^	1.76 × 10^9^ * ± 2.19 × 10^6^	1.15 × 10^9^ * ± 1.44 × 10^6^	2.56 × 10^8^ * ± 3.20 × 10^5^	8.51 × 10^8^ * ± 1.06 × 10^6^	5.37 × 10^8^ * ± 6.68 × 10^5^	6.26 × 10^8^ * ± 7.79 × 10^5^
48 h/2	9.29 × 10^7^ * ±1.16 × 10^5^	2.80 × 10^9^ * ± 3.50 × 10^6^	6.29 × 10^8^ * ± 7.88 × 10^5^	5.75 × 10^8^ * ± 7.18 × 10^5^	1.50 × 10^8^ ± 1.88 × 10^5^	7.62 × 10^8^ * ± 9.53 × 10^5^	3.99 × 10^8^ * ± 4.00 × 10^6^
72 h/2	3.60 × 10^7^ * ± 4.65 × 10^4^	1.93 × 10^9^ * ± 2.49 × 10^6^	5.90 × 10^8^ * ± 7.68 × 10^5^	8.99 × 10^7^ * ± 1.17 × 10^5^	8.26 × 10^7^ * ± 1.03 × 10^5^	9.08 × 10^8^ * ± 1.08 × 10^6^	3.77 × 10^8^ * ± 4.50 × 10^5^
12 h/3 **	5.08 × 10^6^ * ± 8.04 × 10^4^	2.61 × 10^8^ * ± 4.14 × 10^5^	1.28 × 10^9^ * ± 2.03 × 10^6^	1.90 × 10^8^ * ± 3.01 × 10^5^	8.99 × 10^7^ ± 1.42 × 10^5^	3.17 × 10^7^ * ± 5.00 × 10^4^	3.47 × 10^8^ * ± 5.47 × 10^5^
24 h/3	1.28 × 10^8^ * ± 2.03 × 10^5^	9.76 × 10^8^ * ± 1.55 × 10^6^	1.47 × 10^9^ * ± 2.33 × 10^6^	2.85 × 10^8^ * ± 4.52 × 10^5^	8.99 × 10^8^ * ± 1.43 × 10^6^	3.93 × 10^7^ * ± 5.89 × 10^4^	4.75 × 10^8^ * ± 7.50 × 10^5^
48 h/3	3.58 × 10^7^ * ± 5.68 × 10^4^	7.97 × 10^8^ * ± 1.26 × 10^6^	3.34 × 10^8^ * ± 5.28 × 10^5^	1.09 × 10^8^ * ± 1.72 × 10^5^	9.84 × 10^7^ * ± 1.56 × 10^5^	4.19 × 10^7^ ± 6.64 × 10^4^	5.80 × 10^8^ * ± 9.20 × 10^5^
72 h/3	1.85 × 10^7^ * ± 2.93 × 10^4^	6.59 × 10^8^ * ± 1.05 × 10^6^	2.75 × 10^8^ * ±4.38 × 10^5^	3.33 × 10^7^ * ± 5.30 × 10^4^	2.68 × 10^6^ ± 4.27 × 10^3^	2.17 × 10^7^ ± 3.49 × 10^4^	3.00 × 10^8^ * ± 4.80 × 10^5^
12 h/4 **	1.34 × 10^6^ * ± 1.68 × 10^4^	1.77 × 10^7^ * ±2.23 × 10^5^	5.16 × 10^8^ * ± 6.51 × 10^5^	6.59 × 10^7^ ± 8.31 × 10^4^	5.09 × 10^7^ ± 6.43 × 10^4^	4.16 × 10^7^ * ± 5.24 × 10^4^	4.75 × 10^8^ * ± 5.93 × 10^5^
24 h/4	9.21 × 10^6^ * ± 1.16 × 10^5^	1.56 × 10^8^ * ± 1.96 × 10^6^	6.00 × 10^8^ * ± 7.50 × 10^5^	1.25 × 10^8^ * ± 1.56 × 10^5^	1.09 × 10^8^ * ± 1.35 × 10^5^	6.46 × 10^7^ * ± 8.03 × 10^4^	5.47 × 10^8^ * ± 6.80 × 10^5^
48 h/4	8.98 × 10^6^ * ± 1.14 × 10^5^	1.21 × 10^8^ * ± 1.54 × 10^6^	9.69 × 10^7^ * ± 1.24 × 10^5^	5.29 × 10^7^ * ± 6.78 × 10^4^	1.95 × 10^8^ * ± 2.49 × 10^5^	8.26 × 10^7^ * ± 1.05 × 10^5^	6.26 × 10^8^ * ± 7.94 × 10^5^
72 h/4	1.07 × 10^6^ ± 1.35 × 10^4^	1.57 × 10^7^ * ± 1.96 × 10^5^	2.39 × 10^7^ * ± 3.15 × 10^5^	1.39 × 10^7^ * ± 1.83 × 10^5^	1.14 × 10^7^ ± 1.49 × 10^5^	7.34 × 10^7^ * ± 9.57 × 10^4^	4.68 × 10^8^ * ± 6.09 × 10^5^
12 h/5 **	1.63 × 10^6^ * ± 2.07 × 10^4^	6.68 × 10^7^ * ± 8.47 × 10^4^	1.23 × 10^8^ * ± 1.56 × 10^5^	7.15 × 10^7^ ± 8.48 × 10^4^	7.24 × 10^7^ ± 8.59 × 10^4^	8.21 × 10^7^ ± 9.71 × 10^4^	6.62 × 10^8^ ± 7.82 × 10^5^
24 h/5	5.08 × 10^6^ * ± 6.45 × 10^4^	4.94 × 10^8^ ± 6.28 × 10^5^	2.30 × 10^8^ * ± 2.92 × 10^5^	2.17 × 10^8^ ±2.77 × 10^5^	3.75 × 10^8^ ± 4.78 × 10^5^	1.64 × 10^8^ ± 2.08 × 10^5^	9.08 × 10^8^ ± 1.20 × 10^6^
48 h/5	2.60 × 10^7^ ± 3.45 × 10^4^	4.08 × 10^8^ ± 5.42 × 10^5^	3.58 × 10^8^ * ± 4.73 × 10^5^	1.68 × 10^8^ ± 2.21 × 10^5^	1.30 × 10^8^ ± 1.71 × 10^5^	1.11 × 10^8^ * ± 1.46 × 10^5^	7.59 × 10^8^ ± 1.00 × 10^6^
72 h/5	1.73 × 10^7^ * ± 2.30 × 10^4^	3.41 × 10^8^ ± 4.52 × 10^5^	1.05 × 10^8^ ± 1.39 × 10^5^	8.21 × 10^7^ ± 1.09 × 10^5^	2.19 × 10^7^ ± 2.91 × 10^4^	2.52 × 10^7^ ± 3.32 × 10^4^	5.39 × 10^8^ * ± 7.18 × 10^5^
12 h/6 **	2.05 × 10^8^ * ± 2.73 × 10^5^	1.39 × 10^9^ * ±1.86 × 10^6^	1.03 × 10^9^ * ± 1.38 × 10^6^	4.89 × 10^8^ * ± 6.54 × 10^5^	1.41 × 10^9^ * ± 1.89 × 10^6^	1.27 × 10^8^ * ± 1.70 × 10^5^	6.05 × 10^8^ * ± 8.10 × 10^5^
24 h/6	2.30 × 10^8^ * ± 3.06 × 10^5^	2.49 × 10^9^ * ± 3.32 × 10^6^	1.63 × 10^9^ * ± 2.17 × 10^6^	7.39 × 10^8^ * ± 9.84 × 10^5^	1.81 × 10^9^ * ± 2.41 × 10^6^	4.49 × 10^8^ * ± 5.99 × 10^5^	6.93 × 10^8^ * ± 9.24 × 10^5^
48 h/6	7.44 × 10^7^ * ±9.90 × 10^4^	3.30 × 10^9^ * ±4.38 × 10^6^	1.26 × 10^9^ * ± 1.67 × 10^6^	1.90 × 10^8^ ± 2.53 × 10^5^	8.01 × 10^8^ * ± 1.07 × 10^6^	9.32 × 10^8^ * ± 1.24 × 10^6^	2.76 × 10^8^ * ± 3.68 × 10^5^
72 h/6	3.68 × 10^7^ * ±4.90 × 10^4^	2.42 × 10^9^ * ± 3.22 × 10^6^	1.39 × 10^8^ * ± 1.86 × 10^5^	7.38 × 10^7^ * ± 9.85 × 10^4^	7.35 × 10^8^ * ± 9.83 × 10^5^	1.32 × 10^8^ * ± 1.75 × 10^5^	1.73 × 10^8^ * ± 2.31 × 10^5^

* P—*Faecalibacterium prausnitzii* DSM 107840; Ba—*Bifidobacterium animalis* DSM 10140; Bl—*Bifidobacterium longum subs.longum* DSM20219; Lh—*Lactobacillus helveticus* DSM 20075; GG—*Lactobacillus rhamnosus* GG ATCC 53103; Ls—*Lactobacillus salivarius* LA 302; 9—*Lactobacillus plantarum* 299v; ** 0—control; 1—SFE extract + dextran; 2—SFE extract + inulin; 3—SFE extract + trechalose; 4—dextran; 5—inulin; 6—trechalose.

**Table 7:** Assessment of the reduction in cannabinoid concentration after 72 h of cultivation in obtained prebiotic systems 1–3.

	P *	Ba *	Bl *	GG *	Lh *	Ls *	9 *
CBD/1 **	−3.45%	−4.67%	−2.89%	−3.12%	−1.23%	−2.34%	−4.56%
CBG/1	−2.56%	−3.78%	−4.01%	−1.12%	−4.56%	−3.45%	−1.56%
CBN/1	−1.78%	−2.01%	−3.23%	−4.45%	−2.67%	−1.78%	−3.12%
THC/1	−4.01%	−1.12%	−2.34%	−3.56%	−3.78%	−4.01%	−2.56%
CBC/1	−5.12%	−4.23%	−1.34%	−2.45%	−1.56%	−2.67%	−3.78%
The sum of cannabinoids/1	−3.23%	−2.34%	−3.45%	−1.56%	−4.01%	−3.12%	−4.23%
CBD/2 **	0.45%	−4.12%	−3.34%	−2.56%	−1.78%	−2.01%	−3.23%
CBG/2	−1.56%	−3.23%	−2.45%	−1.67%	−3.34%	−4.45%	−2.78%
CBN/2	−2.78%	−2.89%	−3.12%	−4.23%	−2.34%	−1.45%	−3.56%
THC/2	−3.90%	−1.45%	−4.56%	−3.01%	−4.23%	−3.67%	−1.78%
CBC/2	−4.12%	−3.78%	−1.56%	−2.67%	−2.45%	−2.34%	−4.45%
The sum of cannabinoids/2	−2.56%	−4.45%	−2.01%	−1.78%	−3.23%	−1.12%	−3.34%
CBD/3 **	−1.23%	−2.45%	−3.67%	−4.89%	−2.01%	−1.12%	−3.34%
CBG/3	−4.56%	−1.78%	−2.90%	−3.12%	−3.99%	−4.21%	−2.43%
CBN/3	−3.45%	−2.01%	−1.23%	−2.34%	−4.56%	−3.67%	−1.78%
THC/3	−2.90%	−1.12%	−4.21%	−1.45%	−2.56%	−2.78%	−4.01%
CBC/3	−1.34%	−4.45%	−3.78%	−4.90%	−1.11%	−1.23%	−3.89%
The sum of cannabinoids/3	−4.89%	−2.67%	−3.89%	−2.01%	−3.34%	−4.45%	−2.56%

* P—*Faecalibacterium prausnitzii* DSM 107840; Ba—*Bifidobacterium animalis* DSM 10140; Bl—*Bifidobacterium longum subs.longum* DSM20219; Lh—*Lactobacillus helveticus* DSM 20075; GG—*Lactobacillus rhamnosus* GG ATCC 53103; Ls—*Lactobacillus salivarius* LA 302; 9—*Lactobacillus plantarum* 299v. ** 1—SFE extract + dextran; 2—SFE extract + inulin; 3—SFE extract + trechalose.

## Data Availability

The data are contained within the article or Supplementary Materials.

## Supplementary Materials

The following supporting information can be downloaded at: https://www.mdpi.com/article/10.3390/molecules29153574/s1, Figure S1. Sample chromatogram for an SFE extract; Figure S2. Examples of chromatograms of cannabinoid standards used in the developed HPLC method; Table S1. HPLC method validation parameters; Figure S3. Standard curve for the conversion of antioxidant activity in the FRAP assay for Trolox; Figure S4. Standard curve for the conversion of antioxidant activity in the CUPRAC assay for Trolox; Figure S5. Standard curve for the conversion of antioxidant activity in the DPPH assay for Trolox; Figure S6. Standard curve for the conversion of antioxidant activity in the ABTS assay for Trolox; Figure S7. Acetylcholinesterase Inhibition Standard Curve for Galantamine; Figure S8. Butyrylcholinesterase Inhibition Standard Curve for Galantamine; Table S2. Numerical data for the antioxidant activity of the tested hemp extracts; Table S3. Numerical data for the neuroprotective activity of the tested hemp extracts; Table S4. Standard deviation value for assessment of the reduction in cannabinoid concentration after 72 h of cultivation in obtained prebiotic systems 1–3.
