# Supplementary material for: Development of Delivery Systems with Prebiotic and Neuroprotective Potential of Industrial-Grade Cannabis sativa L
Source: Molecules. 2024 Jul 29;29(15):3574. doi: 10.3390/molecules29153574 (PMC11314201; doi:10.3390/molecules29153574)
Supplement: Supplementary file 1 [file molecules-29-03574-s001.zip › molecules-3084972-supplementary.pdf]

Industrial-grade *Cannabis sativa* as a source of neuroprotective active compounds

Szymon Sip [1], Anna Stasiłowicz-Krzemień [1], Anna Sip [2], Piotr Szulc [3], Małgorzata Neumann [3], Aleksandra Kryszak [4] and Judyta Cielecka-Piontek [1,4,*]

[1] Department of Pharmacognosy and Biomaterials, Faculty of Pharmacy, Poznań University of Medical Sciences, Rokietnicka 3, 60-806 Poznań, Poland; szymonsip@ump.edu.pl (S.S.); astasilowicz@ump.edu.pl (A.S.-K.)
[2] Department of Biotechnology and Food Microbiology, Poznan University of Life Sciences, Wojska Polskiego 48, 60-627 Poznań, Poland; anna.sip@up.poznan.pl
[3] Department of Agronomy, Poznań University of Life Sciences, Dojazd 11, 60-632 Poznań, Poland; piotr.szulc@up.poznan.pl (P.S.); malgorzata.neumann@up.poznan.pl (M.N.)
[4] Department of Pharmacology and Phytochemistry, Institute of Natural Fibres and Medicinal Plants, Wojska Polskiego 71b, 60-630 Poznan, Poland; aleksandra.kryszak@iwnirz.pl
* Correspondence: jpiontek@ump.edu.pl

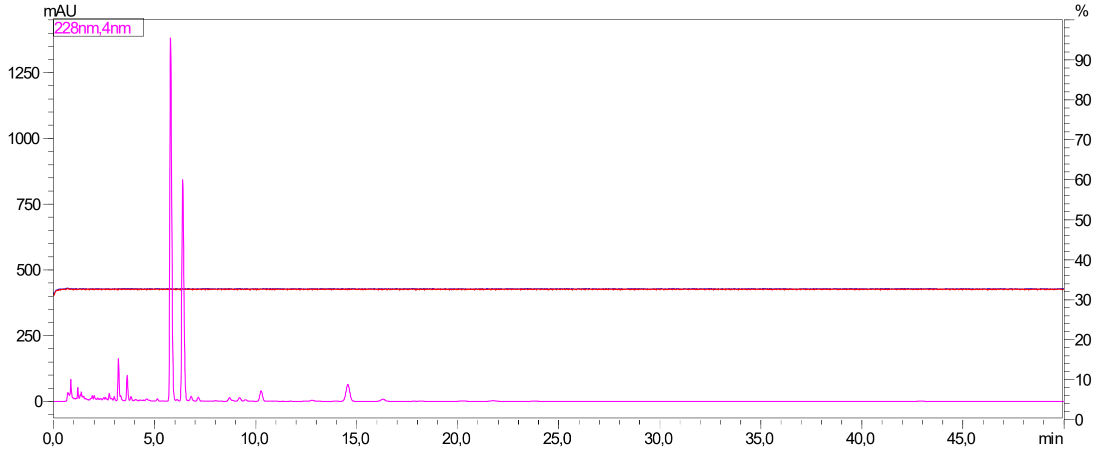

Figure S1. Sample chromatogram for an SFE extract

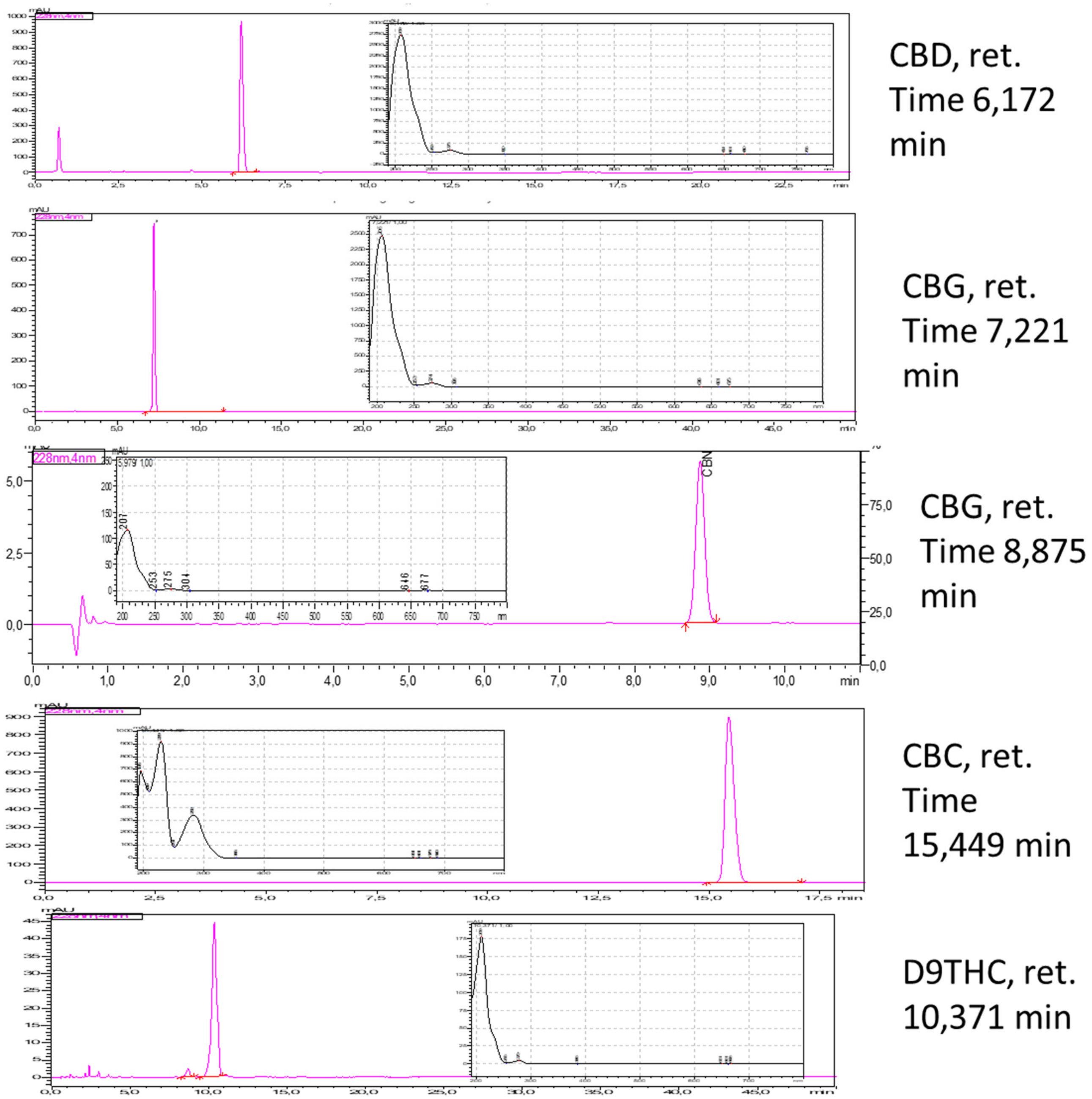

Figure S2. Examples of chromatograms of cannabinoid standards used in the developed HPLC method.

Table S1. HPLC method validation parameters

Parameter	CBD	CBG	CBN	THC	CBC
Linearity: y = ax + b	y= 11662160.52 + 36260.91	Y=11666803.3 – 1071.30	Y=9550243.94 + 1464.75	y=10685545.85 – 6625.09	y=25430597.58 – 3665.13
Correlation coefficient (*r*)	0.9999	0.9999	0.9998	0.9999	0.9997
Range of linearity [mg/ml]	0.001-1.00	0.001-1.00	0.001-1.00	0.001-0.1	0.001-1.00
Limit of detection (LOD): [mg/ml]	1.1512 x 10^{-4}	1.1112 x 10^{-4}	1.2545 x 10^{-4}	1.3212 x 10^{-4}	1.1025 x 10^{-4}
Limit of quantification (LOQ): [mg/ml]	3.7989 x 10^{-4}	3.6669 x 10^{-4}	4.1401 x 10^{-4}	4.3599 x 10^{-4}	3.6383 x 10^{-4}
Intra-day precision. RSD					
0.001 mg/ml	1.12 %	1.09 %	1.11 %	1.19 %	1.10 %
0.05 mg/ml	1.13 %	1.01 %	1.15 %	1.39 %	1.21 %
0.1 mg/ml	1.12 %	1.44 %	1.13 %	1.12 %	1.17 %
Inter-day precision. RSD					
0.001 mg/ml	1.15 %	1.31 %	1.07 %	1.01 %	1.19 %
0.05 mg/ml	1.21 %	1.21%	1.09 %	1.07 %	1.14 %
0.1 mg/ml	1.75 %	1.15 %	1.06 %	1.12 %	1.17 %
Accuracy	99.49 %	99.53 %	99.67 %	99.79 %	99.69 %

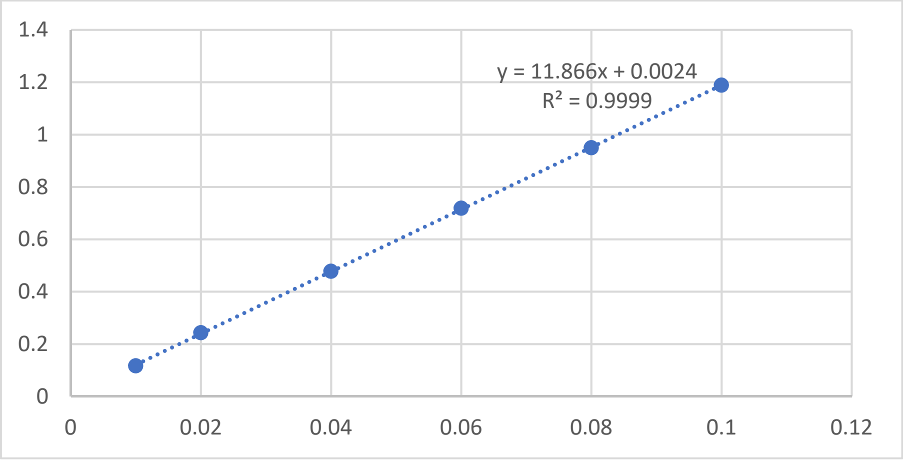

Figure S3. Standard curve for the conversion of antioxidant activity in the FRAP assay for Trolox

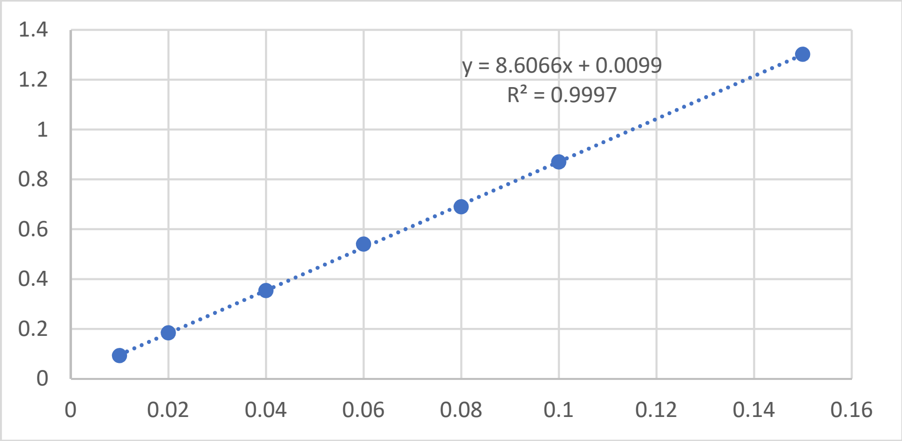

Figure S4. Standard curve for the conversion of antioxidant activity in the CUPRAC assay for Trolox

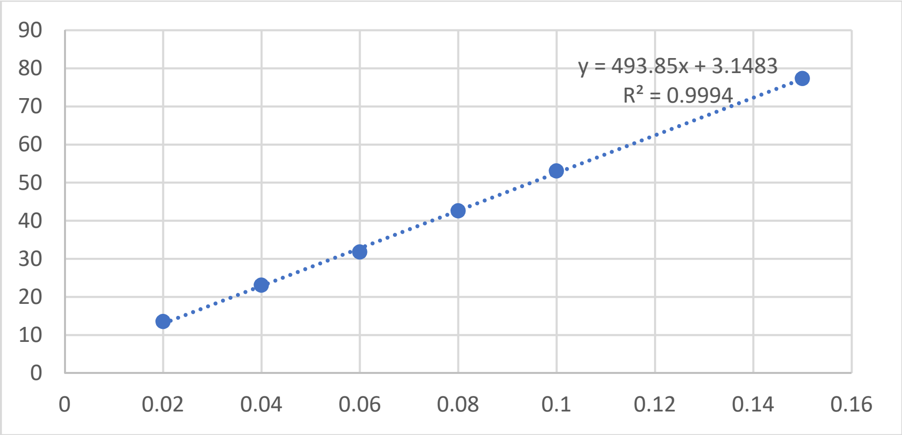

Figure S5. Standard curve for the conversion of antioxidant activity in the DPPH assay for Trolox

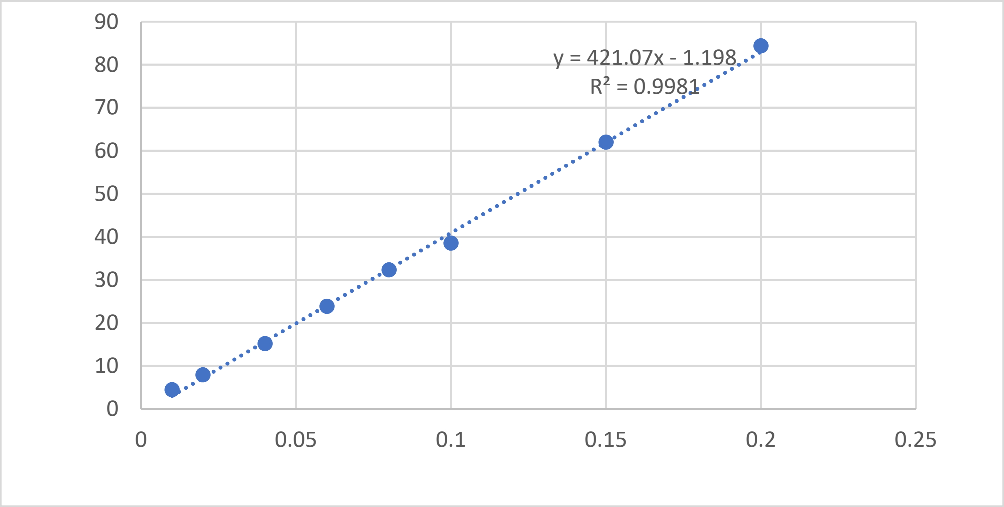

Figure S6. Standard curve for the conversion of antioxidant activity in the ABTS assay for Trolox

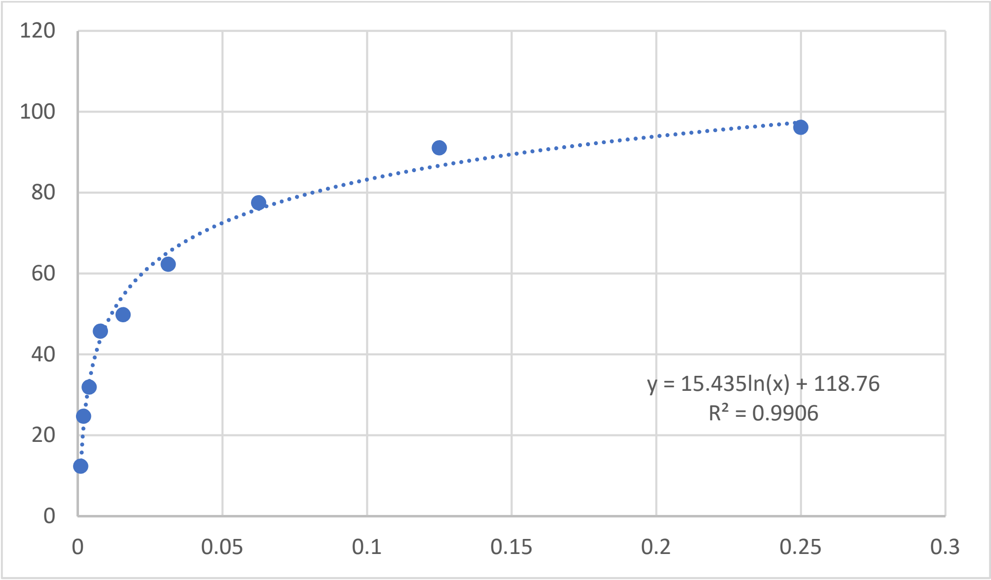

Figure S7. Acetylcholinesterase Inhibition Standard Curve for Galantamine

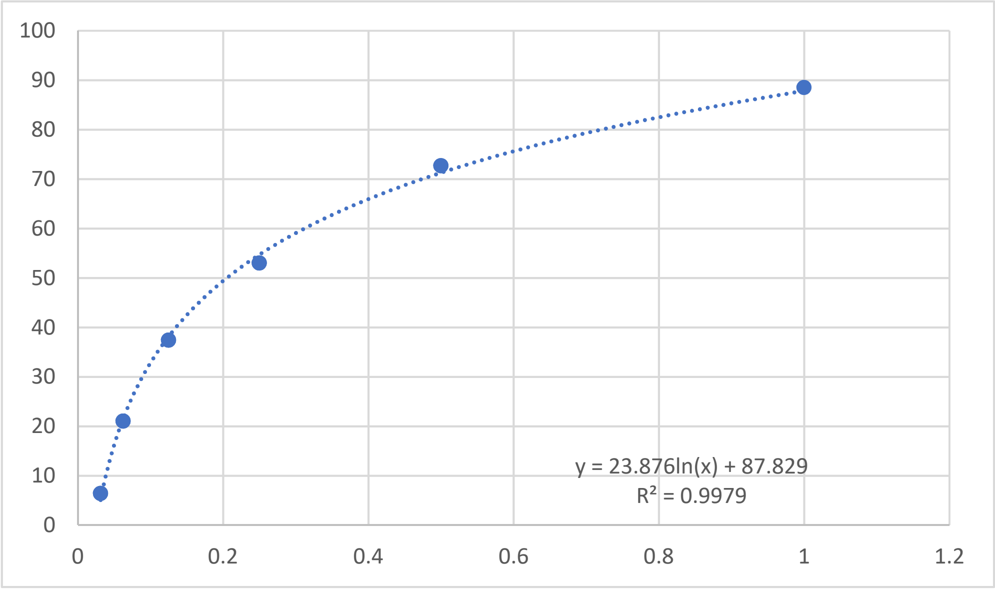

Figure S8. Butyrylcholinesterase Inhibition Standard Curve for Galantamine

Table S2. Numerical data for the antioxidant activity of the tested hemp extracts.

Extract	FRAP	std	CUPRAC	std	DPPH	std	ABTS	std
				Trolox equivalent [mg/ml]				
1	0.025	0.005	0.0333	0.004	0.1153	0.007	0.0219	0.005
2	0.0599	0.005	0.0747	0.004	0.1391	0.006	0.0808	0.004
3	0.0556	0.004	0.0616	0.005	0.1314	0.005	0.0612	0.006
4	0.0531	0.003	0.0584	0.005	0.1316	0.006	0.061	0.004
5	0.0564	0.006	0.0587	0.004	0.1284	0.007	0.0633	0.007
6	0.0196	0.005	0.0218	0.003	0.09859	0.004	0.0109	0.005
7	0.028	0.004	0.0155	0.004	0.08374	0.005	0.0065	0.006
8	0.0505	0.007	0.0343	0.004	0.09928	0.004	0.0291	0.006
9	0.0735	0.005	0.0774	0.005	0.13995	0.003	0.0833	0.004

Table S3. Numerical data for the neuroprotective activity of the tested hemp extracts.

Extract	AchE	std	BChE	std
		Galantamine equivalent [mg/ml]		
1	0.03124	0.00156	0.25447	0.01216
2	5.56E-04	2.78E-05	0.095	0.00454
3	0.0094	4.70E-04	0.563	0.02691
4	0.016	8.00E-04	0.3549	0.01696
5	0.01198	5.99E-04	0.66039	0.03157
6	7.24E-04	3.62E-05	0.14611	0.00698
7	6.36E-04	3.18E-05	0.10334	0.00494
8	0.0124	6.70E-04	0.3129	0.01735
9	0.2438	0.00502	0.89414	0.04274

Table S4. Standard deviation value for assessment of the reduction in cannabinoid concentration after 72 hours of cultivation in obtained prebiotic systems 1-3.

	P*	Ba*	Bl*	GG*	Lh*	Ls*	9*
CBD/1**	± 0.25%	± 0.28%	± 0.21%	± 0.22%	± 0.09%	± 0.17%	± 0.33%
CBG/1	± 0.19%	± 0.28%	± 0.29%	± 0.08%	± 0.33%	± 0.25%	± 0.11%
CBN/1	± 0.13%	± 0.15%	± 0.23%	± 0.32%	± 0.20%	± 0.13%	± 0.22%
THC/1	± 0.29%	± 0.08%	± 0.17%	± 0.26%	± 0.28%	± 0.29%	± 0.19%
CBC/1	± 0.37%	± 0.31%	± 0.10%	± 0.18%	± 0.11%	± 0.20%	± 0.28%
Sum of cannabinoids /1	± 0.24%	± 0.17%	± 0.25%	± 0.11%	± 0.29%	± 0.22%	± 0.31%
CBD/2**	± 0.03%	± 0.30%	± 0.24%	± 0.19%	± 0.13%	± 0.15%	± 0.23%
CBG/2	± 0.11%	± 0.23%	± 0.18%	± 0.12%	± 0.24%	± 0.32%	± 0.20%
CBN/2	± 0.20%	± 0.21%	± 0.23%	± 0.30%	± 0.17%	± 0.10%	± 0.26%
THC/2	± 0.28%	± 0.10%	± 0.33%	± 0.22%	± 0.30%	± 0.26%	± 0.13%
CBC/2	± 0.30%	± 0.28%	± 0.11%	± 0.19%	± 0.18%	± 0.17%	± 0.32%
Sum of cannabinoids /2	± 0.19%	± 0.32%	± 0.15%	± 0.13%	± 0.23%	± 0.08%	± 0.24%
CBD/3**	± 0.09%	± 0.18%	± 0.27%	± 0.35%	± 0.15%	± 0.08%	± 0.24%
CBG/3	± 0.33%	± 0.13%	± 0.21%	± 0.23%	± 0.29%	± 0.30%	± 0.18%
CBN/3	± 0.25%	± 0.15%	± 0.09%	± 0.17%	± 0.33%	± 0.27%	± 0.13%
THC/3	± 0.21%	± 0.08%	± 0.30%	± 0.10%	± 0.19%	± 0.20%	± 0.29%
CBC/3	± 0.10%	± 0.32%	± 0.28%	± 0.35%	± 0.08%	± 0.09%	± 0.28%
Sum of cannabinoids /3	± 0.35%	± 0.20%	± 0.28%	± 0.15%	± 0.24%	± 0.32%	± 0.19%

* P - *Faecalibacterium prausnitzii* DSM 107840; Ba - *Bifidobacterium animalis* DSM 10140; Bl - *Bifidobacterium longum subs.longum* DSM20219; Lh - *Lactobacillus helveticus* DSM 20075; GG - *Lactobacillus rhamnosus* GG ATCC 53103; Ls - *Lactobacillus salivarius* LA 302; 9 - *Lactobacillus plantarum* 299v
1 – SFE extract + dextran; 2 – SFE extract + inulin; 3 – SFE extract + trechalose
